# Optimal Control of FSBB Converter with Aquila Optimizer-Based PID Controller

**DOI:** 10.3390/mi15101277

**Published:** 2024-10-21

**Authors:** Luoyao Ren, Dazhi Wang, Yupeng Zhang

**Affiliations:** College of Information Science and Engineering, Northeastern University, Shenyang 110819, China; loyaren@163.com (L.R.); zhangyupengex@163.com (Y.Z.)

**Keywords:** neural network, adaptive control, four-switch buck–boost, zero voltage switch

## Abstract

This paper presents a new methodology for determining the optimal coefficients of a PID controller for a four-switch buck–boost (FSBB) converter. The main objective of this research is to improve the performance of FSBB converters by fine-tuning the parameters of the PID controller using the newly developed Aquila Optimizer (AO). PID controllers are widely recognized for their simple yet effective control in FSBB converters. However, to further improve the efficiency and reliability of the control system, the PID control parameters must be optimized. In this context, the application of the AO algorithm proves to be a significant advance. By optimizing the PID coefficients, the dynamic responsiveness of the system can be improved, thus reducing the response time. In addition, the robustness of the control system is enhanced, which is essential to ensure stable and reliable operation under varying conditions. The use of AOs plays a key role in maintaining system stability and ensuring the proper operation of the control system even under challenging conditions. In order to demonstrate the effectiveness and potential of the proposed method, the performance of the AO-optimized PID controller was compared with that of PID controllers tuned by other optimization algorithms in the same test environment. The results show that the AO outperforms the other optimization algorithms in terms of dynamic response and robustness, thus validating the efficiency and correctness of the proposed method. This work highlights the advantages of using the Aquila Optimizer in the PID tuning of FSBB converters, providing a promising solution for improving system performance.

## 1. Introduction

Non-isolated DC-DC converters are extensively utilized in industrial and residential power systems due to their numerous advantages, such as shorter design cycles, enhanced reliability, and ease of being upgraded. Among the various voltage lift converter topologies, the four-switch buck–boost (FSBB) converter is particularly notable for its low electrical stress on components, making it a popular choice in voltage-lifting DC-DC applications. Its capability to operate efficiently across a broad range of input and output voltage levels makes it especially suitable for diverse applications in industrial systems and renewable energy technologies [1,2,3]. However, despite its advantages, the FSBB converter inherently presents significant challenges in control due to its nonlinear characteristics and complex dynamics. This nonlinearity arises from the switching nature of the converter, which introduces discontinuities into the system dynamics, coupled with variations in load and input voltage. These factors make it difficult to maintain a stable and efficient output, especially under varying operating conditions. Furthermore, the FSBB converter exhibits multi-modal behavior, meaning that its dynamics can change drastically depending on the mode of operation (buck, boost, or buck–boost), further complicating the control process [4,5,6].

To achieve reliable performance, FSBB converters require an efficient closed-loop feedback control system capable of stabilizing the output voltage and current under dynamic conditions. PID (Proportional–Integral–Derivative) controllers have traditionally been employed in FSBB converters due to their simplicity, ease of implementation, and established performance in regulating DC-DC converters. However, PID controllers face several critical limitations when applied to FSBB converters, particularly in handling the converter’s nonlinear and time-varying behavior [7,8]. While PID control offers satisfactory performance in linear systems, its effectiveness diminishes in nonlinear environments like that of the FSBB converter, where optimal tuning of the proportional, integral, and derivative gains becomes challenging.

The primary limitation of the PID controller in FSBB converters lies in its fixed-gain structure, which does not adapt well to the converter’s varying operating conditions. A fixed-gain PID controller may perform well at a particular operating point but fail to provide satisfactory performance across the converter’s entire operational range. For instance, in the presence of large load changes or input voltage fluctuations, the system may experience overshoot, prolonged settling times, or even instability if the PID gains are not appropriately tuned. Conventionally, these parameters are tuned through trial and error or classical methods such as that of Ziegler–Nichols, often resulting in suboptimal performance, especially in systems with complex dynamics [9]. As a result, there has been growing interest in using metaheuristic optimization algorithms to automatically and efficiently tune PID parameters, improving system response and overall performance. Bio-inspired optimization algorithms are computational methods that draw inspiration from natural processes, such as evolution, swarm behavior, and immune responses, to solve complex optimization problems. These algorithms, including genetic algorithms (GAs), particle swarm optimization (PSO), and Ant Colony Optimization (ACO), mimic the adaptive and self-organizing characteristics of biological systems to explore and exploit search spaces efficiently. By leveraging mechanisms such as mutation, crossover, and selection in GAs, or social interaction and information sharing in PSO, bio-inspired algorithms demonstrate significant potential in handling nonlinear, multimodal, and high-dimensional optimization problems. Their flexibility and robustness make them suitable for a wide range of applications, from engineering design to machine learning. However, they often face challenges such as premature convergence and computational complexity, leading to ongoing research in hybridizing and enhancing these algorithms to improve performance. For instance, Xiao proposes an improved genetic algorithm for PID controller parameter tuning in a beer-filling machine’s liquid level control, demonstrating an enhanced convergence speed, precision, and optimal value attainment compared to other modified algorithms, resulting in improved system responsiveness and the elimination of steady-state error [10]. Similarly, Seyed Mohammad Hossein Mousakazemi presents a genetic algorithm (GA)-tuned PID controller for nuclear power plant control, evaluating various objective functions (IAE, ISE, ITAE, ITSE) and demonstrating that ISE and ITSE offer superior steady-state performance and robustness compared to empirically tuned PID controllers [11]. Arfan Ali Nagra proposes an improved particle swarm optimization algorithm (SIW-APSO-LS) that combines self-inertia weight adaptation with a gradient-based local search strategy to balance exploration and exploitation, demonstrating superior performance over other PSO variants in benchmark optimization tests [12]. A hybrid algorithm (HPSO) based on the combination of particle swarm optimization (PSO) and Simulated Annealing (SA) has been proposed to optimize PID controller parameters, enhancing the global search capability and convergence speed of the system to achieve faster response times and reduce overshooting [13]. Liu presents a modified Crow Search Algorithm (GCSA) using a group strategy and adaptive mechanism to enhance its optimization efficiency by balancing exploration and exploitation. This algorithm demonstrates superior performance over other metaheuristic algorithms through extensive benchmarking and engineering problem solving [14]. Du proposes a reformative Artificial Bee Colony (RABC) algorithm for PID parameter optimization, demonstrating an improved search accuracy, speed, and control performance compared to PSO-PID, DE-PID, and GA-PID in benchmark functions and typical system tests [15]. Zhang and Kong introduce an enhanced Whale Optimization Algorithm (EWOA) with a ranking-based mutation operator to optimize PID controller parameters in an AVR system, demonstrating an improved convergence speed, precision, and stability compared to other algorithms [16]. The use of optimization algorithms to find optimal PID parameters has been a topic of interest for academics. The Aquila Optimizer (AO), a newly developed bio-inspired optimization algorithm, presents a promising solution [17]. The AO is designed to simulate the cooperative hunting strategies of the Aquila bird species, making it well suited for solving complex, nonlinear optimization problems. This paper proposes an innovative approach that applies the AO algorithm to optimize the PID coefficients of an FSBB converter. By doing so, the dynamic response and robustness of the control system are significantly improved, which is essential for maintaining system stability under various operating conditions.

The following sections of this paper are structured as follows: Section 2 analyzes the operation and mathematical model of the FSBB converter. Section 3 details the optimization of the controller using the Aquila Optimizer. Section 4 presents the results of simulation and experimental studies of the closed-loop control. Finally, Section 5 provides a summary of this study’s findings.

## 2. FSBB Converter Operating Mode Analysis and Modeling

### 2.1. Review of FSBB Converter Operation Modes

**Figure 1:**
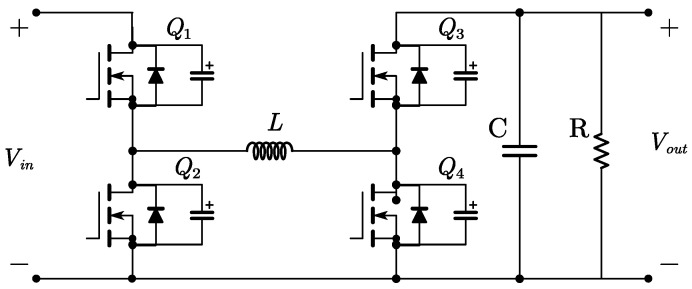
The FSBB converter circuit.

The FSBB circuit schematic is shown in Figure 1. When operating in buck mode, *Q*_1_ and *Q*_2_ conduct complementarily to each other, defining the duty cycle of *Q*_1_ as *D*_1_. When operating in boost mode, *Q*_3_ and *Q*_4_ conduct complementarily to each other, defining the duty cycle of *Q*_4_ as *D*_2_. The relationship between input voltage *V_in_* and output voltage *V_out_* can be obtained from the inductor volt–second balance relationship:

(1) $$V_{out}=\frac{D_{1}}{1-D_{2}}V_{in}$$

**Figure 2:**
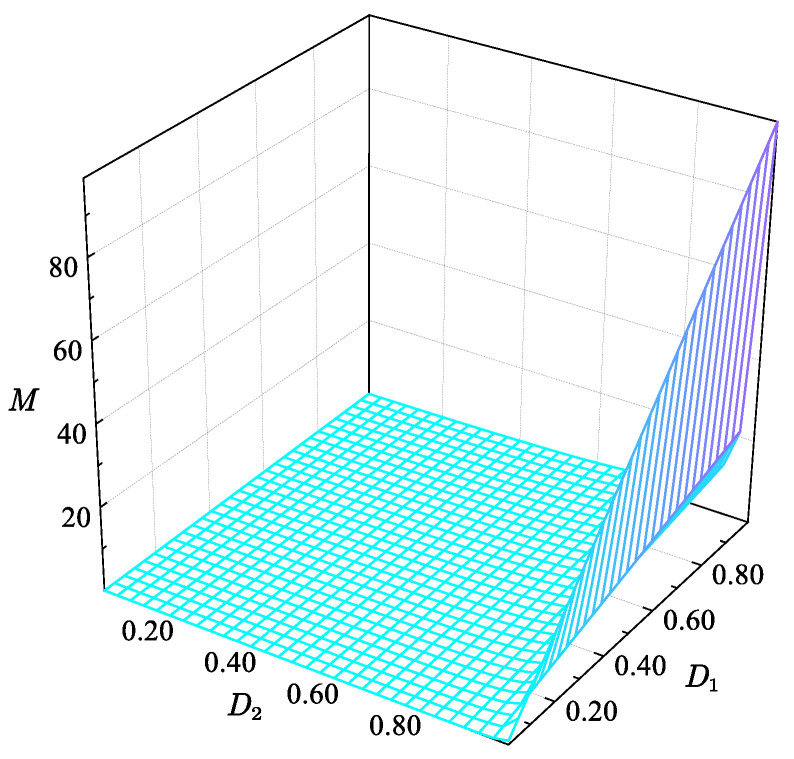
FSBB controller surface gain diagram.

The two mutually independent control degrees of freedom of the FSBB converter, *D*_1_ and *D*_2_, can be observed in Equation (1), which enable the optimal design and control of the converter. The relationship between the FSBB converter gain and duty cycle is illustrated in Figure 2. When the duty cycle is varied between 0.01 and 0.99, the converter gain ranges from 0.01 to 0.99.

When the buck unit of the FSBB converter employs PWM (Pulse Width Modulation), the duty cycle of the boost unit is fixed at 0, indicating that switch *Q*_4_ is continuously deactivated while *Q*_3_ is continually activated. Consequently, the FSBB converter functions as a synchronous rectifier buck converter. Conversely, when the boost unit of the FSBB converter utilizes PWM modulation, the duty cycle of the buck unit is maintained at 1, signifying that switch *Q*_1_ is perpetually activated and *Q*_2_ is consistently deactivated. In this scenario, the FSBB converter operates as a synchronous rectifier boost converter. Thus, *Q*_2_ remains deactivated, making the FSBB converter equivalent to a synchronous rectifier boost converter.

**Figure 3:**
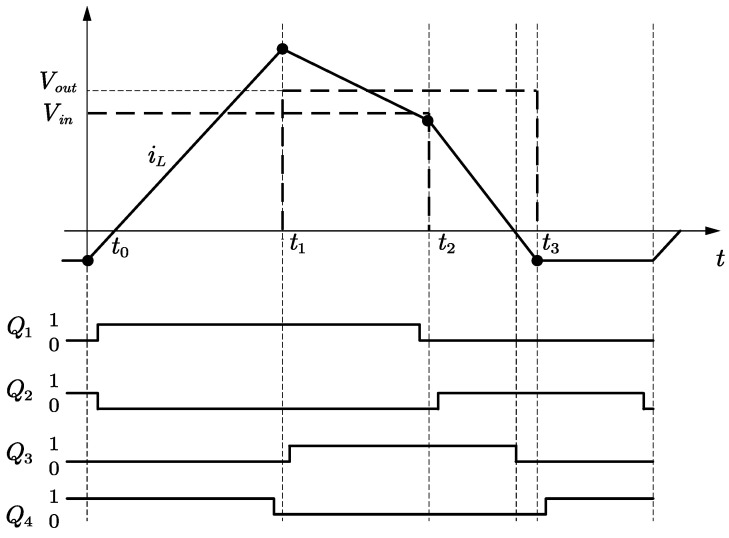
ZVS FSBB control main operating waveforms.

Considering the junction capacitance of the MOSFETs (Metal–Oxide–Semiconductor Field-Effect Transistors), the turn-off process of the four MOSFET switches in the FSBB can be approximated as a zero-voltage turn-off. For an FSBB converter employing the synchronous control of paired MOSFETs, the turn-off of *Q*_1_ and *Q*_4_ results in the discharge of the junction capacitance of *Q*_2_ and *Q*_3_. This is followed by the conduction of the body diodes of *Q*_2_ and *Q*_3_, which clamp the drain-source voltages of *Q*_2_ and *Q*_3_ to a zero-voltage state. This enables *Q*_2_ and *Q*_3_ to achieve zero-voltage turn-on. However, upon the deactivation of *Q*_2_ and *Q*_3_, the inductor current is unable to discharge the junction capacitance of *Q*_1_ and *Q*_4_, thereby preventing zero-voltage turn-on in these components. If the inductor current in *Q*_2_ and *Q*_3_ can reverse, a modest negative current will allow the discharge of the junction capacitance of *Q*_1_ and *Q*_4_, thereby facilitating zero-voltage turn-on. At this point, the synchronous control of the transistors can enable soft switching for all switches in the FSBB converter. Similarly, this method can facilitate soft switching in the FSBB converter when operating in both buck and boost modes. The principal operational waveforms are illustrated in Figure 3.

The buck and boost units are phase-staggered, establishing a direct power path and reducing current ripple through the inductor. The four switches are activated sequentially in accordance with the zero-voltage switching (ZVS) principle. This reduces the junction capacitance that needs to be discharged, thereby facilitating soft switching. One disadvantage of this control mode is the introduction of an inductor current loop phase into the circuit. The modal analysis is presented below for the case where *V_in_* < *V_out_*.

**Figure 4:**
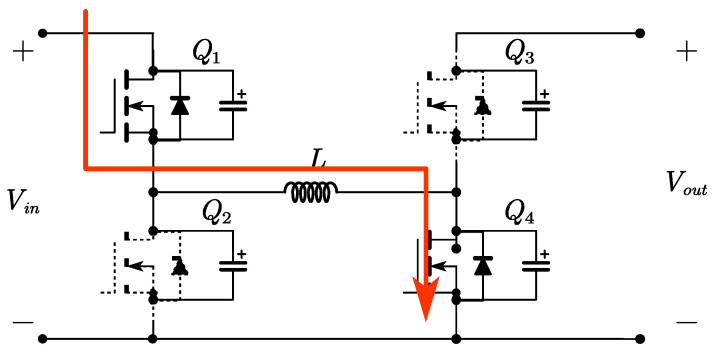
Circuit diagram for time period *t*_0_~*t*_1_.

Time period *t*_0_~*t*_1_: At time *t*_0_, switch *Q*_1_ is turned on with zero voltage, while switch *Q*_4_ remains on. The inductor on the input side begins to store energy, with a voltage across it equal to *V_in_*. The inductor current *i_L_* increases linearly. As shown in Figure 4.

**Figure 5:**
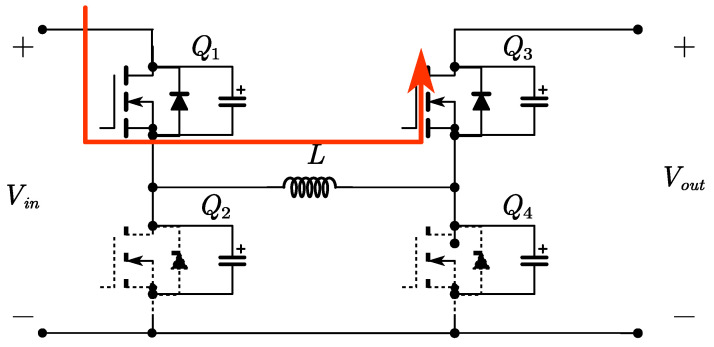
Circuit diagram for time period *t*_1_~*t*_2_.

Time period *t*_1_~*t*_2_: At time *t*_1_, switch *Q*_3_ is turned on with zero voltage, while switch *Q*_1_ remains conducting. The input side directly transfers energy to the output side through the inductor, with a voltage across the inductor of *V_in_*-*V_out_*. The inductor current *i_L_* decreases linearly. As shown in Figure 5.

**Figure 6:**
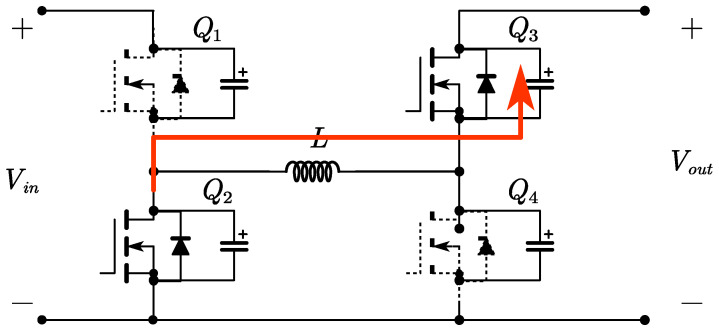
Circuit diagram for time period *t*_2_~*t*_3_.

Time period *t*_2_~*t*_3_: At time *t*_2_, switch *Q*_2_ is turned on with zero voltage, and switch *Q*_3_ remains conducting. The inductor transfers energy to the output side, with a voltage across it equal to *V_out_*. The inductor current *i_L_* decreases linearly. When *i_L_* decreases to zero, switch *Q*_3_ turns off, achieving zero voltage switching (ZVS) and zero current switching (ZCS). As shown in Figure 6.

**Figure 7:**
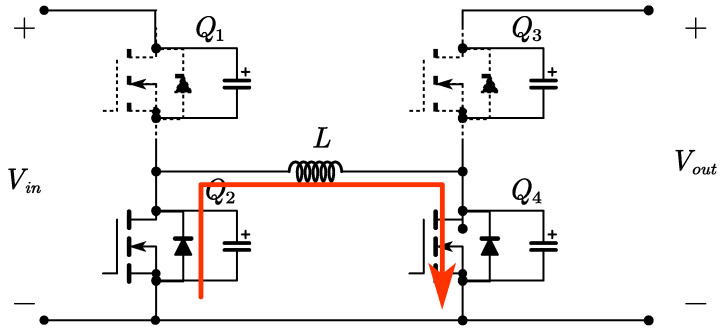
Circuit diagram for time period *t*_3_~*t*_4_.

Time period *t*_3_~*t*_4_: At time *t*_3_, switch *Q*_4_ is turned on with zero voltage, and switch *Q*_2_ remains conducting. The inductor is shorted, allowing a small reverse current to circulate, with a voltage across the inductor equal to zero. The inductor current *i_L_* remains constant. As shown in Figure 7.

The described switching sequence outlines the operation of a power conversion system with four switches (*Q*_1_, *Q*_2_, *Q*_3_, and *Q*_4_). The inductor alternates between energy storage, energy transfer, and current circulation phases, ensuring efficient energy transfer and minimizing stress on the switches.

### 2.2. Mathematical Modeling of FSBB Converter

**Figure 8:**
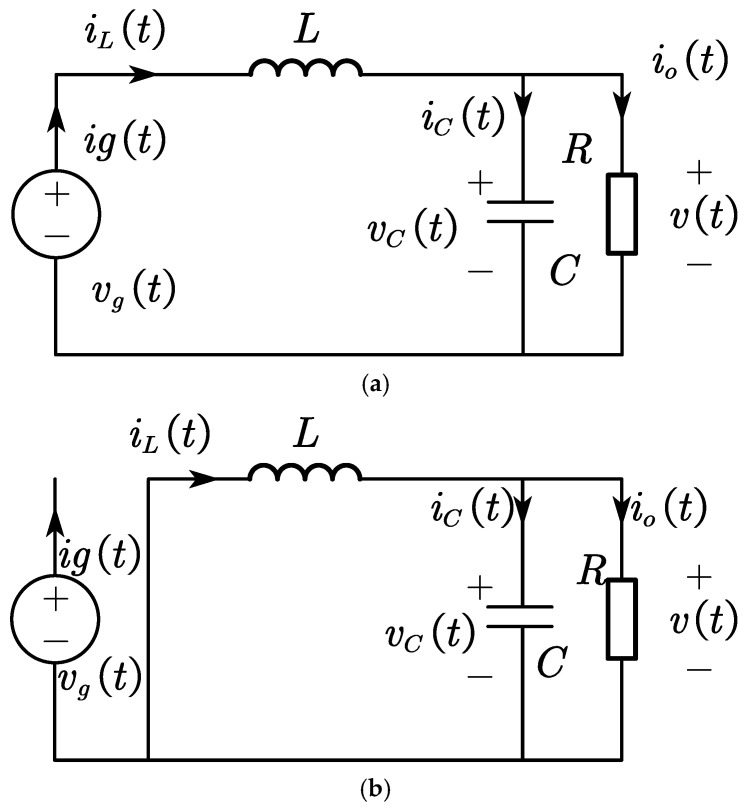
Buck mode equivalent circuit diagram. (**a**) Equivalent circuit diagram for duty cycle stage. (**b**) Equivalent circuit diagram for non-duty cycle stage.

In order to ensure the stability of the output voltage in response to changes in the input voltage or load size, it is necessary to implement a closed-loop feedback mechanism to regulate the duty cycle of the converter within the FSBB system. In order to achieve closed-loop control of the system over a wide input range, small-signal modeling is required for each of the three modes. The equivalent circuit diagrams of the duty cycle stage and non-duty cycle stage of the buck converter are provided in Figure 8 for reference.

The inductor current $i_{L}$(*t*) and the capacitor voltage $v_{C}$(*t*) are used as two independent state variables, the input voltage $v_{C}$(*t*) is used as the input vector, and the output voltage v(*t*) and the input current $i_{g}$(*t*) are used as the output vectors.

The state equations and output equations for the duty cycle stage are as follows:

(2) $$\left\{\begin{array}{c} K\dot{x}\left(t\right)=A_{1}x\left(t\right)+B_{1}u\left(t\right)\\ y\left(t\right)=C_{1}x\left(t\right)+E_{1}u\left(t\right) \end{array}\right.$$

where$\;x\left(t\right)=\left[\begin{array}{c} i_{L}(t)\\ v_{C}(t) \end{array}\right]$, $y\left(t\right)=\left[\begin{array}{c} v(t)\\ i_{g}(t) \end{array}\right]$, $K=\left[\begin{array}{cc} L & 0\\ 0 & C \end{array}\right]$, $A_{1}=\left[\begin{array}{cc} 0 & -1\\ 1 & -\frac{1}{R} \end{array}\right]$, $B_{1}=\left[\begin{array}{c} 1\\ 0 \end{array}\right]$, $C_{1}=\left[\begin{array}{cc} 0 & 1\\ 1 & 0 \end{array}\right]$, $E_{1}=\left[\begin{array}{c} 0\\ 0 \end{array}\right]$.

The state and output equations for the non-duty cycle stage are as follows:

(3) $$\left\{\begin{array}{c} K\dot{x}\left(t\right)=A_{2}x\left(t\right)+B_{2}u\left(t\right)\\ y\left(t\right)=C_{2}x\left(t\right)+E_{2}u\left(t\right) \end{array}\right.$$

where $A_{2}=\left[\begin{array}{cc} 0 & -1\\ 1 & -\frac{1}{R} \end{array}\right]$, $B_{2}=\left[\begin{array}{c} 0\\ 0 \end{array}\right]$, $C_{2}=\left[\begin{array}{cc} 0 & 1\\ 0 & 0 \end{array}\right]$, $E_{2}=\left[\begin{array}{c} 0\\ 0 \end{array}\right]$.

The static operating point equations are as follows:

(4) $$\left\{\begin{array}{c} 0=AX+BU\\ Y=CX+EU \end{array}\right.$$

The duty cycle *D* is brought in to find the static operating point equation:

(5) $$X=\left[\begin{array}{c} \frac{RDV_{g}}{R^{2}}\\ DV_{g} \end{array}\right]$$

(6) $$Y=\left[\begin{array}{c} DV_{g}\\ \frac{RD^{2}V_{g}}{R^{2}} \end{array}\right]$$

Periodic averaging of the space state equations yields an averaged model:

(7) $$K\frac{d{\langle x\left(t\right)\rangle }_{T_{s}}}{dt}dt=\left[d_{1}\left(t\right)A_{1}+d_{2}\left(t\right)A_{2}\right]\;{\langle x\left(t\right)\;\rangle }_{T_{s}}+\left[d_{1}\left(t\right)B_{1}+d_{2}\left(t\right)B_{2}\right]{\langle u\left(t\right)\rangle }_{T_{s}}$$

(8) $${\langle y\left(t\right)\rangle }_{T_{s}}=\left[d_{1}\left(t\right)C_{1}+d_{2}\left(t\right)C_{2}\right]{\langle x\left(t\right)\rangle }_{T_{s}}+\left[d_{1}\left(t\right)E_{1}+d_{2}\left(t\right)E_{2}\right]{\langle u\left(t\right)\rangle }_{T_{s}}$$

**Figure 9:**
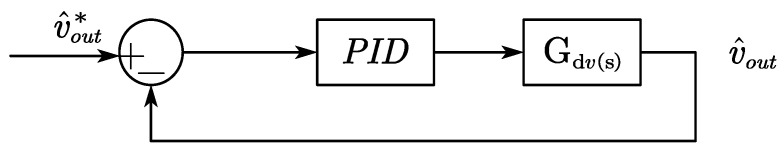
Considered FSBB control structure.

The introduction of small-signal perturbation rewritten as a scalar equation, and the scalar equation of the Laplace change, can be pushed to the buck mode control–output transfer function *G*_*vd*1_(*s*); the same method can be pushed to the boost mode control–output transfer function *G*_*vd*2_(*s*) and buck–boost mode control–output transfer function *G*_*vd*3_(*s*). The focus of this paper, not explained in detail here, directly gives the following transfer function:

(9) $$G_{vd1}\left(s\right)={\left.\frac{\hat{v}\left(s\right)}{\hat{d}\left(s\right)}\right|}_{{\hat{v}}_{g}=0}=\frac{V_{g}}{1+\frac{L}{R}s+LCs^{2}}$$

(10) $$G_{vd2}\left(s\right)={\left.\frac{\hat{v}\left(s\right)}{\hat{d}\left(s\right)}\right|}_{{\hat{v}}_{g}=0}=\frac{V_{g}\left(1-\frac{L}{D_{2}^{2}R}s\right)}{D_{2}^{2}+\frac{L}{R}s+LCs^{2}}$$

(11) $$G_{vd3}\left(s\right)={\left.\frac{\hat{v}\left(s\right)}{\hat{d}\left(s\right)}\right|}_{{\hat{v}}_{g}=0}=\frac{V_{g}\left(1-\frac{L}{D_{2}^{2}R}s\right)}{\frac{D_{2}^{2}}{D_{1}^{2}}+\frac{L}{R}s+LCs^{2}}$$

where *D*_1_ is the input duty cycle and *D*_2_ is the output duty cycle. The control block diagram is presented in Figure 9.

## 3. Aquila Optimizer Optimized PID Controller

### 3.1. PID Controller

The traditional incremental digital PID control algorithm is based on the following formula:

(12) $$\Delta u\left(k\right)=k_{P}\left[e\left(k\right)-e\left(k-1\right)\right]+k_{I}e\left(k\right)+k_{D}\left[e\left(k\right)-2e\left(k-1\right)+e\left(k-2\right)\right]$$

where *k_P_* means the proportional coefficient; *k_I_* means the integral coefficient; and *k_D_* means the differential coefficient.

**Figure 10:**
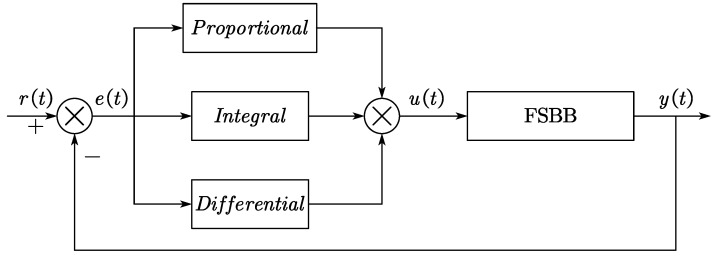
PID controller structure.

Figure 10 offers a visual representation of the PID controller’s structure.

Despite its wide applicability, a conventional PID controller may struggle with nonlinear systems like the FSBB converter, where switching dynamics and load variations introduce nonlinearities. These challenges necessitate the optimization of PID parameters to achieve the desired control performance [18,19].

### 3.2. Aquila Optimizer

The Aquila Optimizer (AO) is a newly developed metaheuristic algorithm inspired by the hunting strategies of the Aquila bird species [17]. It has demonstrated superior performance in solving complex optimization problems due to its effective balance between exploration and exploitation, making it particularly suitable for global optimization tasks. In this study, the AO algorithm is employed to determine the optimal parameters *K_p_*, *K_i_* and *K_d_* of the PID controller, ensuring the optimal performance of the FSBB converter.

The AO algorithm mimics the dynamic hunting behavior of Aquila birds, which alternate between broad searching (exploration) and targeting specific, promising regions (exploitation). The algorithm consists of four main phases corresponding to different hunting strategies used by Aquila birds. These phases ensure that the algorithm can escape local optima and efficiently converge to the global optimum.

**Figure 11:**
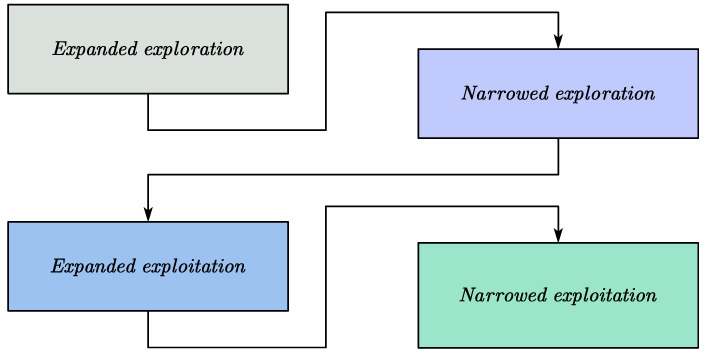
The flowchart of the AO algorithm.

The flowchart of the AO algorithm is shown in Figure 11.

The AO algorithm can be described as follows:

1. Expanded exploration: In the early stages, the AO performs a broad search across the solution space to explore different regions and avoid premature convergence. The position of each solution in the population is updated according to a random search strategy to maintain diversity in the search process. The mathematical representation is as follows:
  (13) $$P_{1}\left(i+1\right)=P_{best}\left(i\right)*\left(1-\frac{i}{I}\right)+\left(P_{M}\left(i\right)-P_{best}\left(i\right)*r_{1}\right)$$
  (14) $$P_{M}\left(t\right)=\frac{1}{n}\sum_{x=1}^{n}P_{x}\left(i\right)$$
  where *P_M_*(*i*) is the average position of all Aquilas in the current iteration, *P*_1_(*I* + 1) is the next iteration’s solution generated by the first search method (*P*_1_), and *P_best_*(*i*) is the best position thus far. Here, *i* and *I* represent the current and maximum iterations, respectively. The population size is denoted by *n*, and *r*_1_ is the random number.
2. Narrowed exploration: As the algorithm progresses, the search focuses on promising regions near the best solutions found so far. This phase refines the search to improve the accuracy of the solutions. The position is updated using the following equation:
  (15) $$P_{2}\left(i+1\right)=P_{best}\left(i\right)*LF\left(D\right)+P_{R}\left(i\right)+\left(b-a\right)*r_{2}$$
  where *P_R_* is the random position, *D* is the dimension size, and *LF* represents Levy flight functions.
3. Expanded exploitation: The AO mimics the high-speed, precise attacks of Aquila birds by intensifying the search around the best solution, allowing the algorithm to converge faster. The position is updated as follows:
  (16) $$P_{3}\left(i+1\right)=\left(P_{best}\left(i\right)-P_{M}\left(i\right)\right)\times \alpha -r_{4}+\left(\left(UB-UL\right)\times r_{5}+LB\right)\times \delta$$
  where *P_best_* (*i*) is the best position so far, *P_M_*(*i*) is the average value of the current population’s positions, and *UB* and *LB* are the upper and lower bounds. *α* and *⸹* are the exploitation adjustment parameters.
4. Narrowed exploitation: In the final phase, the AO fine-tunes the solutions to ensure that the global optimum is achieved with high precision. The position is updated as
  (17) $$\left\{\begin{array}{c} P_{4}\left(i+1\right)=QF\times P_{best}\left(i\right)-\left(G_{1}\times P\left(i\right)\times r_{6}\right)-\left(-G_{2}\times LF\left(D\right)\right)+r_{7}\times G_{1}\\ QF\left(i\right)=i^{\frac{2rand\left(\right)-1}{{\left(1-I\right)}^{2}}}\\ G_{1}=2\times r_{8}-1\\ G_{2}=2\times \left(1-\frac{i}{I}\right) \end{array}\right.$$
  where *P*(*i*) is the current position, *QF* is the quality function value, *G*_1_ is the movement parameter of the Aquilas (in the range of [−1, 1]), and *G*_2_ represents the flight slope while chasing prey, decreasing from 2 to 0. *r*_6_, *r*_7_, and *r*_8_ are the random numbers.

**Figure 12:**
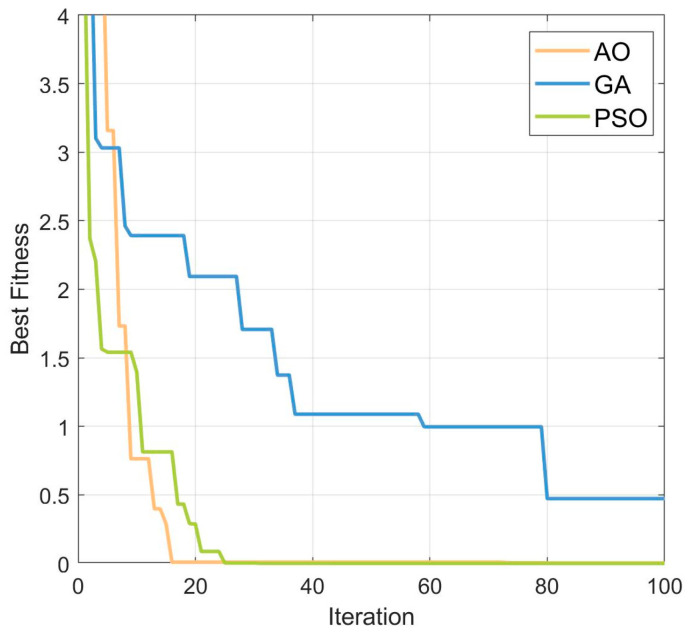
The convergence curves of the three optimization algorithms.

**Figure 13:**
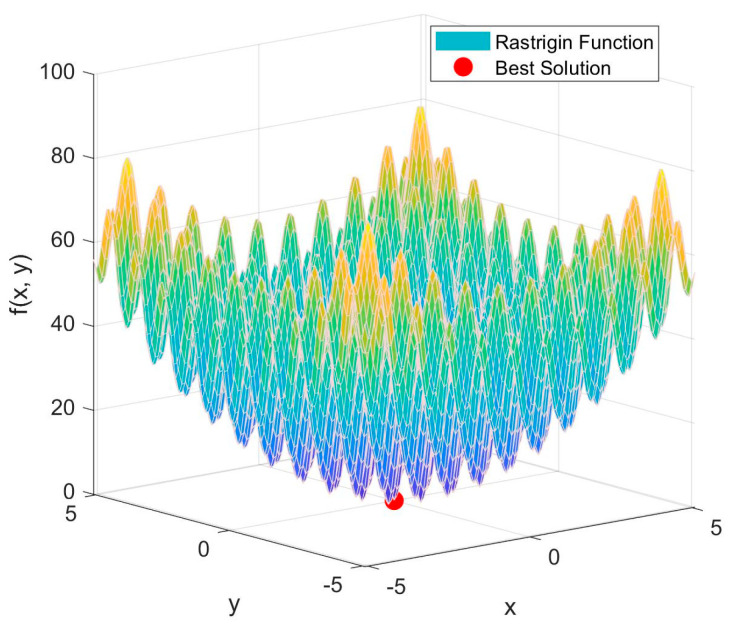
Rastrigin function and best solution.

To verify the superiority of the AO optimization algorithm, the Rastrigin function was optimized using the AO, PSO, and GA algorithms, respectively. Figure 12 shows the convergence curves of the three optimization algorithms, demonstrating how the algorithms gradually find better solutions as the number of iterations increases. The comparison highlights the superiority of the AO algorithm. Figure 13 presents a 3D plot of the Rastrigin function, where the red dots indicate the optimal solutions found by the AO optimization algorithm.

### 3.3. Design of the Proposed Controller

In the proposed control strategy, the AO algorithm is used offline to determine the optimal PID parameters *K_p_*, *K_i_* and *K_d_* for the FSBB converter. Unlike real-time or online tuning methods, where controller parameters are adjusted dynamically during operation, the AO-based optimization is performed prior to system operation. Once the optimal parameters are determined, they are fixed and used for controlling the FSBB converter during normal operation. The offline optimization process using the AO can be summarized as follows:

Initialization: A population of candidate solutions is randomly generated within predefined bounds.

Fitness Evaluation: For each candidate solution, the PID controller’s performance is evaluated using a cost function. The AO algorithm is used to tune the PID parameters by minimizing the integral of time-weighted absolute error (ITAE) as the performance index:

(18) $$ITEA=\int \left|e\left(t\right)\right|*tdt$$

where *e*(*t*) is the error between the desired and actual output.

Optimization: The AO algorithm updates the candidate solutions by balancing the exploration and exploitation phases, continuously improving the PID parameters to minimize the cost function.

Convergence: The algorithm repeats the optimization process until a stopping criterion is met, such as reaching a maximum number of iterations or achieving a satisfactory cost function value.

**Figure 14:**
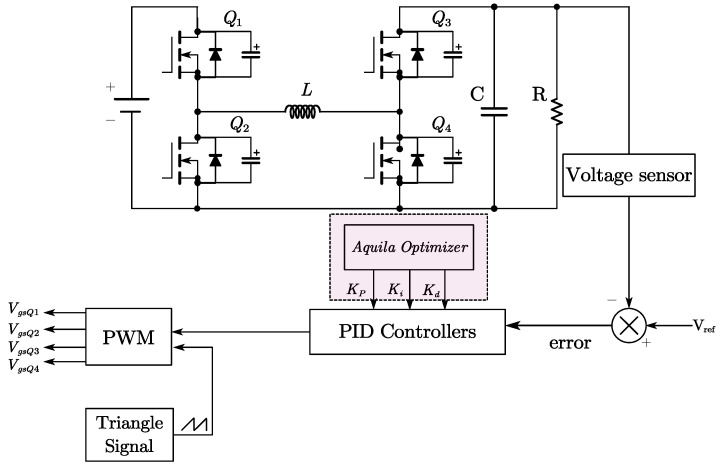
Block diagram representing a proposed controller.

Once the optimal parameters are found, the PID controller is implemented with these fixed values. The proposed controller structure is shown in Figure 14, where the AO algorithm is used to fine-tune the PID parameters offline. These optimized parameters are then applied during the operation of the FSBB converter to ensure optimal performance.

This AO-based offline optimization offers several key advantages. By optimizing the PID parameters, the system’s transient response is significantly improved, resulting in faster settling times and a reduced overshoot. Additionally, the control system’s robustness is enhanced, allowing it to perform effectively under a wide range of operating conditions, including variations in input voltage and load disturbances. Unlike traditional tuning methods, the AO algorithm’s ability to escape local optima ensures that the globally optimal PID parameters are identified, leading to superior overall control performance.

## 4. Results and Discussion

In this chapter, the performance of the proposed Aquila Optimizer (AO)-based PID controller for the four-switch buck–boost (FSBB) converter is analyzed through various simulations. The chapter is divided into several subsections to evaluate the controller’s performance under different operating conditions and to compare it with other optimization techniques. The results for each simulation are discussed in detail, considering key performance factors such as settling time, overshoot, steady-state error, and robustness under uncertainties.

### 4.1. Simulation Under Normal Conditions

**Figure 15:**
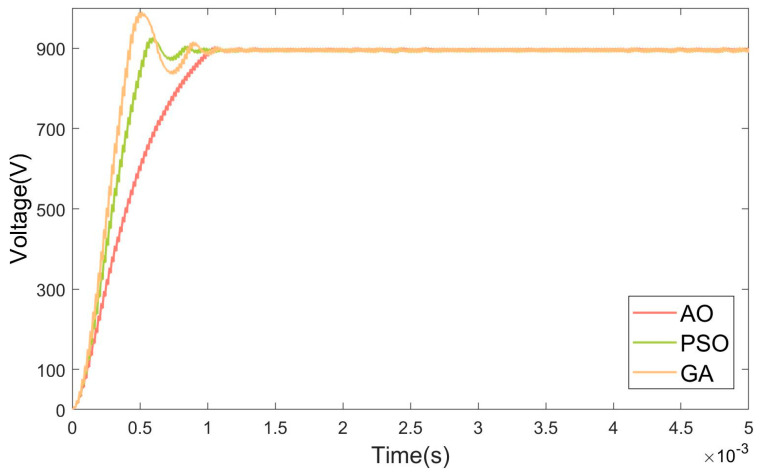
Boost mode simulation result graph.

**Figure 16:**
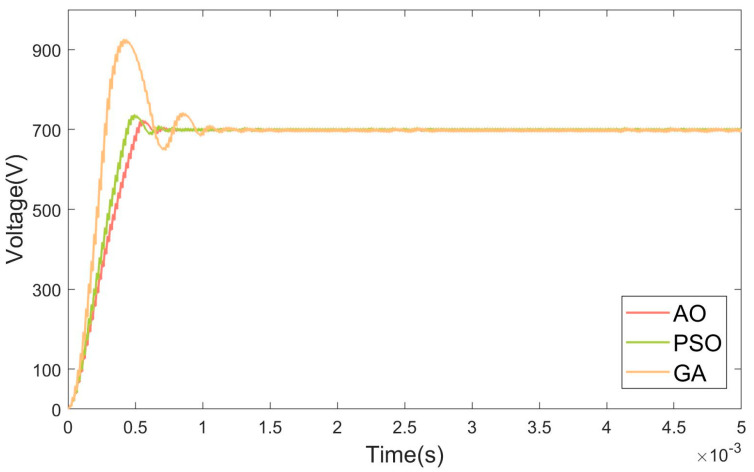
Buck mode simulation result graph.

The objective of this section is to evaluate the basic performance of the AO-optimized PID controller under standard operating conditions, including a typical input voltage, nominal load, and no external disturbances. Two sets of experiments were designed to achieve voltage boosting and bucking, respectively, from 700 V to 900 V and from 900 V to 700 V. The results of these experiments are displayed in Figure 15 and Figure 16.

The experimental results demonstrate that the AO-optimized PID controller significantly outperforms the other optimization algorithms (GA and PSO) in both boost and buck operating modes. In the boost mode, all three controllers (GA, PSO, and AO) reached steady state within 0.001 s. However, the GA-optimized controller exhibited a 9.8% overshoot, and the PSO-optimized controller showed a 2.9% overshoot. In contrast, the AO-optimized controller achieved zero overshoot, indicating a superior transient response and control precision. This result highlights the ability of the AO-optimized controller to maintain stability and avoid excessive overshoot during the boost operation. In the buck mode, the AO-optimized controller reached steady state within 0.001 s, which was 0.0043 s faster than the PSO-optimized controller and 0.0058 s faster than the GA-optimized controller. Regarding overshoot, the GA-optimized controller exhibited a substantial 32% overshoot, while the PSO-optimized controller reduced this to 5%. The AO-optimized controller further minimized overshoot to just 2.7%, demonstrating a more controlled and stable response during the buck operation.

In the buck mode, the AO-optimized controller achieved significantly faster settling times compared to both the GA- and PSO-optimized controllers. The AO-optimized controller consistently exhibited a reduced overshoot in both boost and buck modes, with zero overshoot in the boost mode and minimal overshoot in the buck mode. The AO algorithm enhances the ability of the PID controller to respond quickly and accurately to changes in system dynamics, ensuring a more stable and precise control. The AO algorithm’s ability to efficiently exploit both local and global search regions allows it to optimize the PID parameters with a higher degree of precision. The FSBB converter’s nonlinear dynamics introduce multiple conflicting objectives, such as reducing overshoot while minimizing settling time and maintaining stability under disturbances. The AO’s flexible exploration–exploitation mechanism ensures that it finds a balance between these competing objectives, leading to a more robust and finely tuned control system.

The experimental results show that the AO’s iterative structure allows for adaptive tuning, which is particularly beneficial in systems with significant dynamic behavior, such as FSBB converters. The PID controller’s performance is highly sensitive to parameter variations in nonlinear systems, and small changes can lead to vastly different system behaviors. The AO algorithm dynamically adjusts its search strategies based on the evolving fitness landscape, which enables it to fine-tune the PID parameters more precisely to minimize overshoot and settling time. This adaptability is crucial in managing the fast transients and nonlinearities of FSBB converters, where fixed or static optimization approaches, like GA, may struggle to adapt to changing system conditions or complex error surfaces.

### 4.2. Simulation Under Load Step Changes

**Figure 17:**
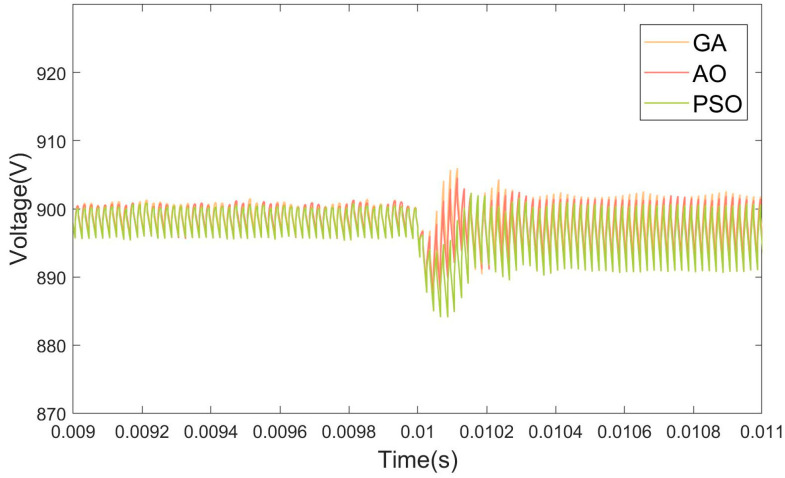
Simulation under load step changes result graph.

This simulation tests the controller’s robustness when subjected to sudden load changes by evaluating its transient response, settling time, and the magnitude of overshoot or undershoot following the load change. Varying the load resistance size at 0.01 s, the simulation results are shown in Figure 17.

The AO-optimized PID controller exhibits significantly enhanced robustness across various operating conditions, particularly in its ability to maintain system stability and performance in the presence of load disturbances. This robustness is reflected in its minimal overshoot and undershoot, ensuring that the system does not deviate excessively from the desired setpoint during transient events. Additionally, the controller demonstrates a remarkably quick recovery time following sudden load changes, allowing the system to swiftly return to its steady state with minimal delay. In comparison to alternative controllers, such as those optimized by genetic algorithms (GAs) or particle swarm optimization (PSO), the AO-optimized PID controller consistently achieves superior performance, effectively minimizing the impact of disturbances and ensuring a smoother, more stable operation. The results confirm that the AO-optimized controller is highly capable of handling varying load conditions, making it a reliable and efficient solution for applications requiring precise and robust control under dynamic conditions.

### 4.3. Simulation Under Input Voltage Variations

**Figure 18:**
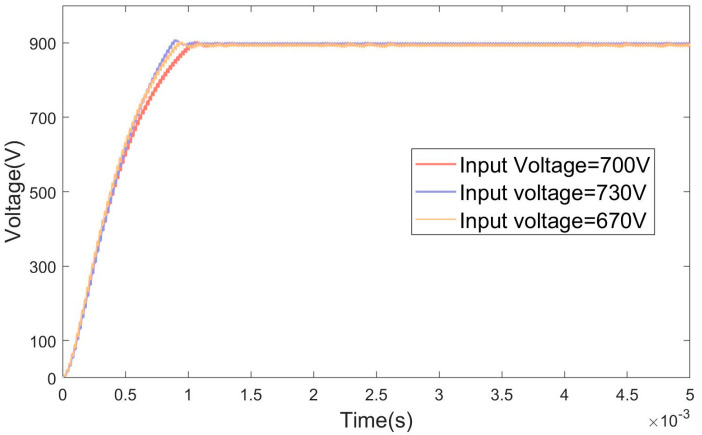
Simulation under input voltage variations result graph.

This section examines the controller’s performance when the input voltage fluctuates, a critical factor for FSBB converters, which are often used in applications where the input supply is unstable. The input voltages were set to 670 V, 700 V, and 730 V. The simulation results are shown in Figure 18.

The AO-optimized PID controller is specifically designed to maintain a stable output voltage with minimal transient deviations, ensuring that any disturbances or changes in system conditions are handled smoothly. It excels in reducing both overshoot and undershoot, leading to a more consistent and accurate performance, even during sudden changes in load or external disturbances. Additionally, the controller exhibits faster recovery times, allowing the system to quickly return to its steady-state operation after transient events. This quick response minimizes the duration of any deviations from the desired setpoint, ensuring that the system remains efficient and stable under a wide range of operating conditions. The combination of minimal transient behavior and rapid recovery makes the AO-optimized PID controller highly reliable for applications that require precise control and stability in dynamic environments.

## 5. Discussion

The results of this study demonstrate the effectiveness of the Aquila Optimizer (AO)-optimized PID controller in enhancing the performance of four-switch buck–boost (FSBB) converters. The AO-optimized controller achieved significantly faster settling times and a reduced overshoot in both boost and buck modes compared to conventional tuning methods. In boost mode, it eliminated overshoot entirely, while in buck mode, it reduced overshoot to just 2.7%, compared to 32% for the GA-optimized controller. This highlights the AO algorithm’s ability to fine-tune PID parameters for improved control precision and dynamic response. The specific results are given in Table 1.

The controller’s robustness was also validated under load step changes and input voltage variations. It maintained system stability with minimal transient deviations and quick recovery times, proving highly capable of handling disturbances and ensuring a consistent performance. The AO’s balance between exploration and exploitation allowed it to find globally optimal PID parameters, unlike traditional methods that often result in suboptimal performance in nonlinear systems like FSBB converters. The specific results are given in Table 2.

In practical applications, the AO-optimized PID controller offers significant benefits for systems requiring stable and efficient control under varying operating conditions. Its ability to minimize energy losses, reduce component stress, and ensure reliable performance makes it a valuable solution for industrial power supplies and renewable energy systems. Future research could explore real-time adaptive control using the AO and its application to other types of converters.

## Figures and Tables

**Table 1:** Comparison of performance of PID controllers in FSBB with different optimizations.

Control Mode	Optimization Algorithms	Transient Duration	Overshoot
Boost Mode	AO	1 ms	0%
	PSO	1 ms	2.9%
	GA	1 ms	9.8%
Buck Mode	AO	1 ms	2.7%
	PSO	4.4 ms	5%
	GA	8.5 ms	32%

**Table 2:** AO-optimized PID controller performance under load and input voltage variation.

Test Conditions	Test Metrics	Test Result
Load step change	System stability	Stable, minimum transient deviation
Input voltage variations	Recovery time	Fast recovery

## Data Availability

Data are contained within the article.
